# Severity related neuroanatomical and spontaneous functional activity alteration in adolescents with major depressive disorder

**DOI:** 10.3389/fpsyt.2023.1157587

**Published:** 2023-04-05

**Authors:** Xiaoliu Zhang, Jun Cao, Qian Huang, Su Hong, Linqi Dai, Xiaorong Chen, Jianmei Chen, Ming Ai, Yao Gan, Jinglan He, Li Kuang

**Affiliations:** ^1^Department of Psychiatry, The First Affiliated Hospital of Chongqing Medical University, Chongqing, China; ^2^Mental Health Center, University-Town Hospital of Chongqing Medical University, Chongqing, China

**Keywords:** thalamus, gray matter, premotor cortex, visual cortex, cortical thickness, anterior cingulate, ALFF, adolescent depression

## Abstract

**Background:**

Major depressive disorder (MDD) is a disabling and severe psychiatric disorder with a high rate of prevalence, and adolescence is one of the most probable periods for the first onset. The neurobiological mechanism underlying the adolescent MDD remains unexplored.

**Methods:**

In this study, we examined the cortical and subcortical alterations of neuroanatomical structures and spontaneous functional activation in 50 unmedicated adolescents with MDD vs. 39 healthy controls through the combined structural and resting-state functional magnetic resonance imaging.

**Results:**

Significantly altered regional gray matter volume was found at broader frontal-temporal-parietal and subcortical brain areas involved with various forms of information processing in adolescent MDD. Specifically, the increased GM volume at the left paracentral lobule and right supplementary motor cortex was significantly correlated with depression severity in adolescent MDD. Furthermore, lower cortical thickness at brain areas responsible for visual and auditory processing as well as motor movements was found in adolescent MDD. The lower cortical thickness at the superior premotor subdivision was positively correlated with the course of the disease. Moreover, higher spontaneous neuronal activity was found at the anterior cingulum and medial prefrontal cortex, and this hyperactivity was also negatively correlated with the course of the disease. It potentially reflected the rumination, impaired concentration, and physiological arousal in adolescent MDD.

**Conclusion:**

The abnormal structural and functional findings at cortico-subcortical areas implied the dysfunctional cognitive control and emotional regulations in adolescent depression. The findings might help elaborate the underlying neural mechanisms of MDD in adolescents.

## 1. Introduction

Major depressive disorder (MDD) is a chronic and debilitating neuropsychiatric disorder affecting populations worldwide and ranking as the leading cause of global disease burden (1). The estimated lifetime risk is from 15% to 18%, and adolescence, specifically mid-adolescence, is one of the most probable periods for the first episode onset. This period is also critically important for adolescent brain development. MDD is fundamentally characterized by depressed mood and anhedonia with a lack of interest and activity. It also includes the emotional symptoms of persistent and pervasive feelings of worthlessness and guilt, and finally could even generate suicide ideation, plan, or attempt. The neurovegetative symptoms include fatigue or loss of energy, poor sleep quality, and decreased appetite and weight. Moreover, the neurocognitive symptoms include the diminished ability to think or concentrate and psychomotor retardation or agitation (2). The point rate of MDD in Chinese children and adolescents was 1.3%, according to a recent meta-analysis (3). Adolescent depression could generate negative outcomes such as poor educational outcomes, social impairment, insomnia, smoking, and substance use. Thus, the examination of neurobiological features is critical for later preventative measures implementation and appropriate mental health services.

Neuroimaging technique, such as magnetic resonance imaging (MRI), has provided a non-invasive way to gain insight into the brain's anatomical structures and neuronal functional activity. Structural MRI offers an effective way to investigate neuroanatomical structures, and the volumetric analyses of gray matter (GM) and surface calculation of cortical thickness (CT) are two main and reliable measurements used for research. In adolescent studies, neuroanatomical alterations at the frontal–limbic circuit have been implicated in the psychopathology of MDD. Specifically, compared to matched controls, adolescents with MDD were found to have regional cortical reductions in frontal areas, such as medial orbitofrontal and superior frontal gyrus, primary and higher order visual, somatosensory, and motor areas according to the study from ENIGMA working group (4). On the contrary, the thicker middle frontal and anterior cingulate cortex were also observed in MDD adolescents compared to controls, suggesting the developmental trajectory of the frontal lobe in adolescent MDD (5). Alongside the frontal lobe, the thinner cortical area at the left occipital area (precuneus and cuneus) was found in first-episode drug-naive adolescents with MDD (6). The temporal lobe morphology alteration of hippocampal and parahippocampal volumes was also reported in adolescent MDD (7–9). Furthermore, smaller volume at other subcortical nuclei, including the amygdala, thalamus, and nucleus accumbens, was found in depressed adolescents (10).

Resting-state functional MRI (rs-fMRI) is also a branch of technique widely used to evaluate brain neuronal activation. There are several rs-fMRI metrics used to reflect the brain functional activity such as the amplitude of low-frequency fluctuation (ALFF) and regional homogeneity (ReHo). ALFF could reflect the intensity of regional spontaneous neuronal activity (11), while ReHo is an indicator of complexity and synchronicity of the neuronal activation (12). Previously, we have successfully applied the ALFF and ReHo analyses to detect the abnormality of neuronal activity in adult depression (13–15). Adolescent studies have suggested that depressive symptoms are associated with aberrant neural processing of reward (16, 17). Moreover, adolescent MDD was reported to have impaired functional activation associated with emotional processing, regulation, and memory (18). Furthermore, the other deficits in developing neural systems, such as visuospatial attention and sustained visual attention, in adolescent MDD patients were also suggested (19, 20).

Considering there is still a lack of consistent findings in adolescent MDD research, which is partly caused by the differences in research sample (i.e., medication) and imaging modality. Therefore, the present study aimed to provide a comprehensive clarification of the neural characteristics of unmedicated adolescent MDD through the application of both structural and functional analyses at the same time. First, we utilized voxel-based morphometry (VBM) to examine the subcortical structure of GM volume in adolescent MDD in comparison with healthy controls (HCs). Second, we used surface-based morphometry (SBM) to examine the CT in MDD patients compared with HC. Third, we also investigated the subcortical ALFF and ReHo as measurements of functional activity in MDD patients compared to HC. Finally, we calculated the surface ALFF and ReHo measurements and compared them between MDD and HC groups. Therefore, we hypothesized that there would be neuroanatomical and functional alterations in cortico-subcortical brain regions in adolescent MDD. Furthermore, we expected those brain morphometry characteristics to be correlated with clinical scores.

## 2. Materials and methods

### 2.1. Participants

A total of 66 adolescents with MDD were recruited from the Department of Psychiatry at the First Affiliated Hospital of Chongqing Medical University, China. The study was approved by the research Ethics Committee of the university. The experimental procedures were explained, and written informed consent was obtained from the adolescents and their caregivers. Inclusion criteria for MDD and HC groups involved the following: adolescents who are right-handed (determined by patient interview and parents' confirmation), aged between 13 and 18 years, with normal intelligence, and also having normal to corrected vision. The Structured Clinical Interview for Diagnostic and Statistical Manual IV (DSM-IV) Axis I disorder (SCID-I) was administered for the diagnosis of depressive disorder by two qualified psychiatrists. Exclusion criteria included adolescents with a psychiatric axis-I co-morbidity (i.e., anxiety disorder), a history of neurological diseases or seizures, a significant head injury or concussion, standard MRI contraindications, a history of substance/alcohol abuse or dependence, a history of psychiatric disorders or suicide among first-degree relatives, and other clinically relevant abnormalities based on the medical history or the laboratory examination. The severity of depression was measured on the day of MRI scanning using the 17-item Hamilton Depression Rating Scale (HDRS). In addition, 40 right-handed HC matched for gender, age, and education were recruited from the local community through advertisements. The subjects were screened with the Structured Clinical Interview for DSM-IV, non-patient edition (SCID-NP). Subjects with a past or current DSM-IV axis-I diagnosis, neurological illness, a history of head trauma with loss of consciousness, and a history of psychiatric disorders or suicidal behavior among first-degree relatives were excluded.

### 2.2. Imaging protocols

All MR images were acquired with a 3.0-T GE Signa HDxt (General Electric Healthcare, Chicago, Illinois, USA) scanner equipped with a standard eight-channel head coil. Contiguous sagittal T1-weighted images across the entire brain were acquired with a fast gradient echo (FGRE) sequence: time of echo (TE) = 3.1 msc, time of repetition (TR) = 8 msc, flip angle = 12°, the field of view (FOV) = 240 mm, voxel size = 0.938 × 0.938 × 1 mm^3^, and no gap.

The rs-fMRI data were obtained using an echo-planar image (EPI) pulse sequence with the following parameters: 33 axial slices, TE = 40 msc, TR = 2,000 msc, in-plane resolution = 64 × 64 pixels, flip angle = 90°, FOV = 240 mm, voxel size = 3.75 × 3.75 × 4 mm^3^, and no gap. A total of 240 time points were obtained over 8 min.

### 2.3. Data processing and analysis

#### 2.3.1. Anatomical image processing

Both anatomical and functional image processings were conducted using DPABISurf, a toolbox offering a user-friendly interface for brain imaging data processing and analysis (21). The toolbox was executed in MATLAB (R2021b). For processing and analysis steps, default parameters following standard protocol were used. The images were resampled to the original size (i.e., 0.938 × 0.938 × 1 mm^3^ for an anatomical image; 3.75 × 3.75 × 4 mm^3^ for a functional image). The T1-weighted (T1w) image was corrected for intensity nonuniformity and used as T1w-reference throughout the workflow. The T1w-reference was then skull-stripped. Furthermore, brain tissue segmentation of cerebrospinal fluid (CSF), white matter (WM), and GM was performed on the brain-extracted T1w.

Brain surfaces were reconstructed using recon-all (FreeSurfer 6.0.1) (22), and the brain mask estimated previously was refined with a custom variation of the method to reconcile the derived segmentations of the cortical GM. Volume-based spatial normalization to one standard space (MNI152NLin2009cAsym) was performed through non-linear registration.

After the anatomical image was preprocessed, the volumetric GM and surface CT were smoothed using a Gaussian filter with a 6-mm full width at half maximum (FWHM).

#### 2.3.2. Functional image processing

For each of the resting-state blood-oxygenation-level dependent (BOLD) signals per subject, the following preprocessing was performed. The first 10 volumes were removed, and then, a reference volume and its skull-stripped version were generated. The BOLD reference was then co-registered with the T1w reference. Head-motion parameters with respect to the BOLD reference are estimated. BOLD runs were slice-time corrected. The BOLD time series were then resampled to surface space (fsaverage5) and original volumetric space. The nuisance covariates of WM, CSF, and global signal as well as head motion were regressed. Principal components are estimated after high-pass filtering the preprocessed BOLD time series for the two component-based noise correction (CompCor) variants: temporal (tCompCor) and anatomical (aCompCor). tCompCor components are then calculated from the top 5% variable voxels within a mask covering the subcortical regions. This subcortical mask is obtained by heavily eroding the brain mask, which ensures it does not include cortical GM regions. For aCompCor, components are calculated within the intersection of the aforementioned mask, and the union of CSF and WM masks is calculated in the T1w space, after their projection to the native space of each functional run.

After the functional images were preprocessed, covariates nuisance was further regressed to acquire the subcortical ALFF/ReHo at volumetric space and cortical ALFF/ReHo at surface space. ALFF corresponds to the mean amplitude low-frequency fluctuation bands (0.01–0.1 Hz), and ReHo represents the homogeneity of a time course for a given voxel relative to that of the time courses of the 26 nearest neighboring voxels. Finally, those two metrics were smoothed through a Gaussian filter with a 6-mm FWHM.

### 2.4. Statistical analysis

Differences in those anatomical and rs-fMRI metrics between MDD patients and HC were assessed using statistical analysis in DPABISurf. To investigate GM volumetric changes in MDD patients, a two-sample *t*-test was used. Age, gender, and total intracranial volume were entered into the general linear model as covariates. An initial voxel-wise threshold was set at a *p*-value of <0.001 with a subsequent family-wise error (FWE) corrected *p*-value of <0.05 at the cluster level. Moreover, differences in the CT between groups were assessed with age and gender entered into the general linear model as covariates. An initial voxel-wise threshold was set at a *p*-value of <0.001. A threshold-free cluster enhancement (TFCE) approach was used to correct for multiple comparisons, and a significance threshold of *q* < 0.025 was used for each hemisphere.

Two-sample *t*-tests were also conducted to investigate the measurements of volumetric and cortical ALFF/ReHo between the MDD and HC groups. Together with the age and gender, the generated mean FD_Jenkinson value was included as covariance to control the head motion effects. An initial voxel-wise threshold was set at a *p-*value of <0.05, and with subsequent FWE corrected *p*-value of <0.05 at the cluster level for volumetric analyses. While for the surface analysis, an initial voxel-wise threshold was set at a *p-*value of <0.05. Still, a TFCE approach was used to correct for multiple comparisons, and a significance threshold of *q* < 0.025 was used for each hemisphere.

Furthermore, we extracted the mean value of GM volume, CT, ALFF, and ReHo of the significant clusters in both MDD and HC. *Post hoc* two-sample *t*-tests were carried out to compare the group differences. Moreover, correlation analyses were carried out to examine the relationship between those structural and functional measurements and clinical scores. The xjView toolbox (https://www.alivelearn.net/xjview) and DPABISurf_VIEW were used for later volumetric and surface imaging results visualization.

## 3. Results

### 3.1. Participants and clinical characteristics

In total, 66 patients with MDD and 40 HC were initially screened for inclusion in the study. A total of 16 MDD patients and one HC were excluded for missing and invalid imaging or demographics data, as well as taking medication, leaving 50 MDD patients (39 female patients/11 male patients, 15.80 ± 1.43 years old) and 39 HC (26 female/13 male patients, 15.82 ± 1.89 years old) to be included into the analysis. One subject with head motion larger than 3 mm translation and a 3-degree rotation was excluded from functional activity analysis. Among the MDD individuals, 96% of individuals were in the first episode of depression, and the average frequency was 1.04. A total of 46 MDD subjects with records of disease course were available, and the average score was 19.94, corresponding to moderate severity. In total, 46 MDD subjects with records, of course, were available, and the average course of MDD was 14.62 months. The demographic and clinical characteristics of these individuals are shown in Table 1.

**Table 1:** Demographic and clinical characteristics of participants.

**Measure (mean, S.D.)**	**MDD (*n* = 50)**	**HC (*n* = 39)**	**Statistics**
**Gender**, ***n***			
Female	39	26	^a^*p* = 0.232
Male	11	13	
Age, years	15.80 (1.43)	15.82 (1.89)	*t* = 0.058, *p* = 0.95
Education, years	9.84 (1.75)	9.92 (2.49)	*t* = 0.19, *p* = 0.85
^*^HDRS^b^	19.94 (5.72)	–	
Age of onset, years	14.77 (2.01)	–	
Course^c^, months	14.62 (13.46)	–	
First episode, *n* (%)	48 (96%)	–	
Frequency	1.04 (0.20)	–	

* HDRS, hamilton depression rating scale.

a Indicates the chi-square test.

b This calculation came from 44 MDD subjects.

c This calculation came from 46 MDD subjects.

### 3.2. Group comparison of GM volume

#### 3.2.1. Increased GM volume in adolescent MDD

**Figure 1:**
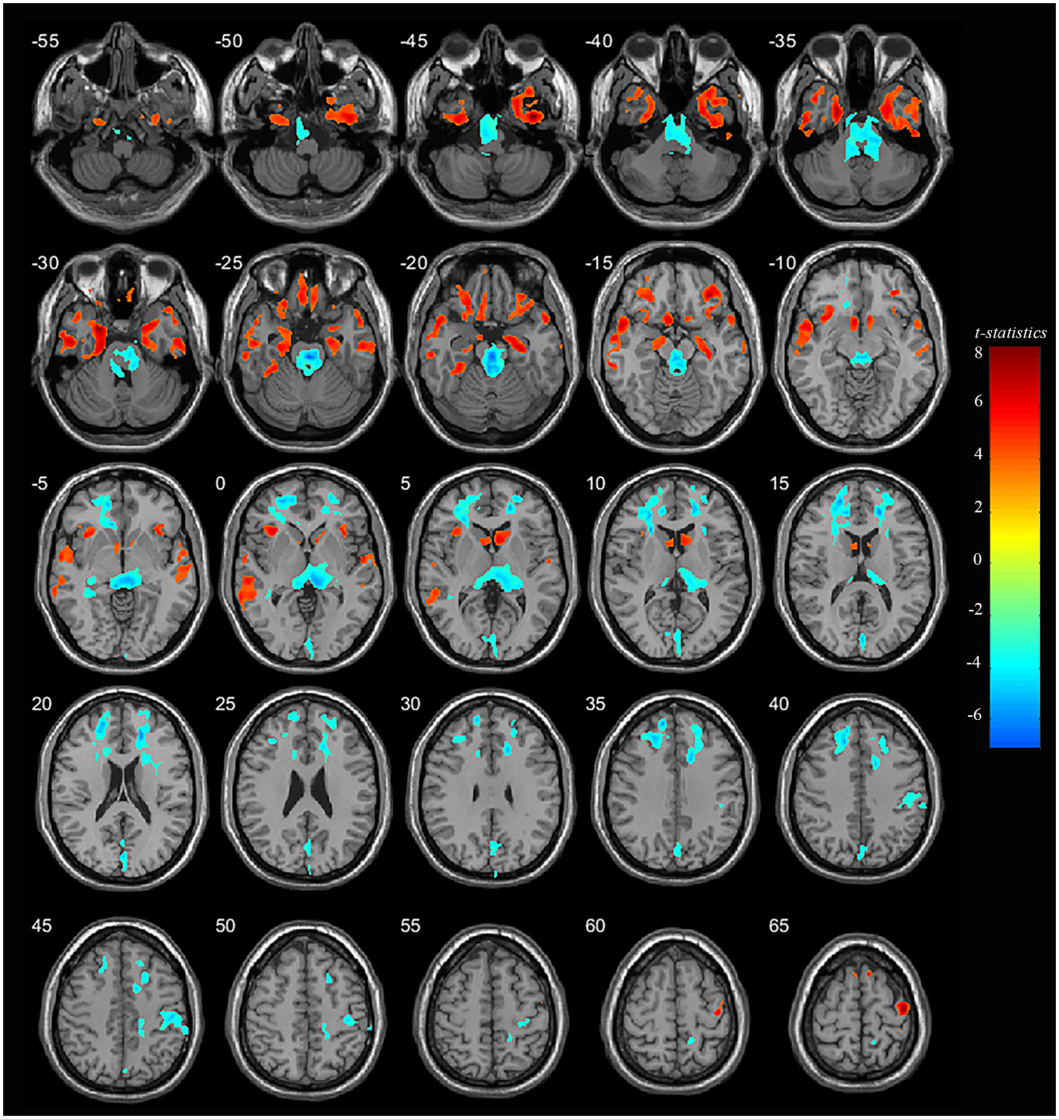
Adolescent MDD showed GM volume disturbances at broader frontal–temporal–parietal and subcortical brain areas. Compared to HC, adolescent MDD had significantly increased GM volume at the right inferior temporal including parahippocampal and fusiform gyrus, left parahippocampus extending to inferior temporal, left mTG, left insula extending to orbIFG, right orbIFG extending to the right insula and left caudate, bilateral paracentral lobule and SMA. Meanwhile decreased value was found at the bilateral thalamus, left SFG and MFG, bilateral cuneus, right ACC and MCC, right postcentral and precentral gyrus. The color bar depicts the t-statistics.

Significant differences were observed in regional GM volume between the MDD and HC groups. Compared to controls, MDD patients showed significantly increased GM volume in the right inferior temporal extending to the right parahippocampus and fusiform gyrus (peak value = 7.92; at [39, −5, −46]; *k* = 27,758 voxels), the left parahippocampus extending to the left hippocampus and fusiform gyrus (peak value = 7.33; at [−25, 3, −30]; *k* = 12,230 voxels), right precentral extending to the paracentral lobule (peak value = 7.09; at [41, −13, 65]; *k* = 13,489 voxels), the right orbital part of the inferior frontal (orbIFG) extending to the right insula and left caudate (peak value = 6.88; at [31, 36, −15]; *k* = 13,834 voxels), the left inferior temporal extending to middle temporal (mTG, peak value = 6.33; at [−56, −7, −32]; *k* = 15,996 voxels), the left paracentral lobule extending to precentral (peak value = 6.39; at [−14, −21, 79]; *k* = 6,487 voxels), the left insula extending to the left orbIFG (peak value = 5.97; at [−35, 21, −1]; *k* = 7,023 voxels), the left supplementary motor area (SMA, peak value = 5.84; at [−9, 12, 71]; *k* = 2,013 voxels), and the right SMA (peak value = 5.19; at [8, 10, 71]; *k* = 1,544 voxels) at a *p*-value of <0.001 (corrected) at cluster level (see Figure 1 and Table 2).

**Table 2:** Locations of regional increased GM volume in the MDD group.

**Region**	**MNI coordinates of peak**			**Peak *t*-value**	**Spatial extent (in contiguous voxels)**
	***x***	***y***	***z***		
*MDD > HC*					
**R. Inferior temporal**	39	−5	−46	7.92	27,758
R. Hippocampus	28	−18	−18	6.30	1,563
**L. ParaHippocampus**	−25	3	−30	7.33	12,230
**R. Precentral**	41	−13	65	7.09	13,489
R. Paracentral lobule	12	−25	80	6.81	1,073
**R. orbIFG**	31	36	−15	6.88	13,834
L. Caudate	−7	11	−12	6.14	1,311
R. Insula	33	22	−3	5.79	1,417
**Inferior temporal**	–−56	−7	−32	6.33	15,996
L. mTG	−60	–−26	1	5.65	7,077
**L. Paracentral lobule**	−14	−21	79	6.39	6,487
L. Precentral	−27	−16	75	5.30	1,108
**L. Insula**	−35	21	−1	5.97	7,023
L. orbIFG	−23	28	−23	5.85	2,735
**L. SMA**	−9	12	71	5.84	2,013
**R. SMA**	8	10	71	5.19	1,544

#### 3.2.2. Decreased GM volume in adolescent MDD

Compared to HC, MDD patients were also found to have significantly decreased GM volume in the right thalamus extending to the left side (peak value = −6.40; at [−1, −22, −23]; *k* = 35,684 voxels), the right anterior cingulate cortex (ACC) extending to the middle cingulate cortex (MCC) (peak value = −6.83; at [17, 40, 18]; *k* = 13,965 voxels), the left superior frontal (SFG) extending to the middle frontal gyrus (MFG) (peak value = −6.35; at [−14, 52, 33]; *k* = 20,335 voxels), right postcentral extending to right supramarginal (peak value = −5.85; at [48, −19, 44]; *k* = 3,601 voxels), and the left cuneus extending to the left calcarine (peak value = −5.36; at [0, −84, 16]; *k* = 6,006 voxels) at a *p*-value of <0.001 (corrected) at the cluster level (see Figure 1 and Table 3).

**Table 3:** Locations of regional decreased GM volume in the MDD group.

**Region**	**MNI coordinates of peak**			**Peak *t*-value**	**Spatial extent (in contiguous voxels)**
	***x***	***y***	***z***		
*MDD < HC*					
**R. Thalamus**	−1	−22	−23	−6.40	35,684
L. Thalamus	−13	−30	−2	−4.05	1,970
**R. ACC**	17	40	18	−6.83	13,965
R. MCC	15	22	32	−5.95	1,372
**L. SFG**	−14	52	33	−6.35	20,335
L. MFG	−22	51	14	−6.22	4,568
**R. Postcentral**	48	−19	44	−5.85	3,601
R. Supramarginal	60	−32	42	−4.49	685
**L. Cuneus**	0	−84	16	−5.36	6,006
L. Calcarine	1	−92	1	−4.79	2,471

### 3.3. Group comparison of CT

#### 3.3.1. Decreased CT in adolescent MDD at the left hemisphere

**Figure 2:**
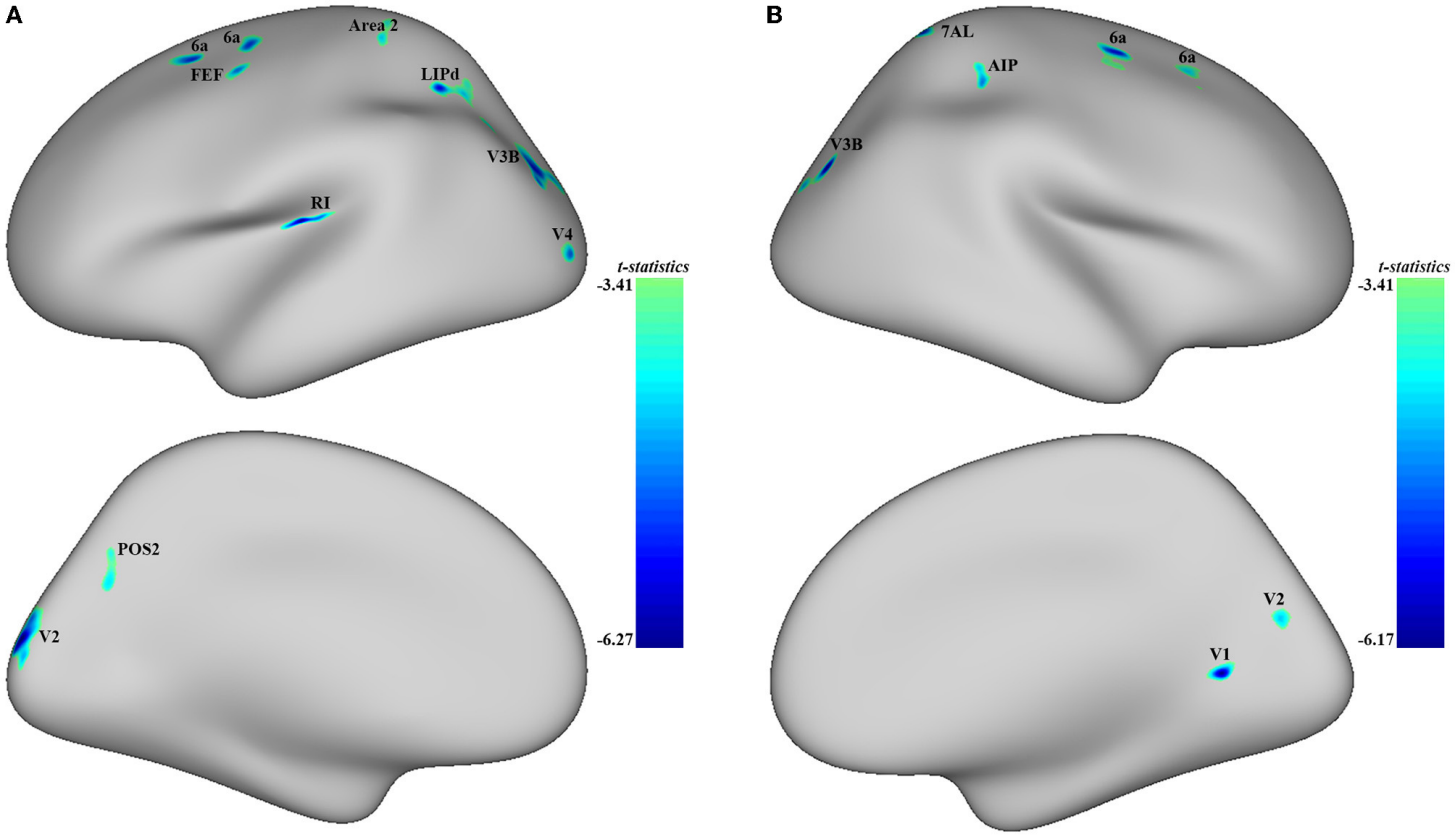
Adolescent MDD showed significantly decreased CT at both the left and right hemispheres. **(A)** Adolescent MDD showed significantly decreased CT value at several clusters including the LIPd (superior parietal cortex), V3B (dorsal stream visual cortex), V2 and V4 (early visual cortex), RI (early auditory areas), POS2 (posterior opercular cortex), and 6a and FEF (premotor cortex) at the left hemisphere. The color bar depicts the *t*-statistics; **(B)** Adolescent MDD showed a significantly decreased CT value at several clusters including the 6a (superior premotor subdivision), V3B (dorsal stream visual cortex), AIP and 7AL (superior parietal cortex), V1 (primary visual cortex), and V2 (early visual cortex) at the right hemisphere. The color bar depicts the t-statistics.

Significant differences were observed in regional CT between the MDD and HC groups at both the left and right hemispheres. At left side, MDD patients showed a significantly decreased CT in the ***LIPd*** (peak value *t* = −5.70; at [−5, −53, 42]; *k* = 852 vertices), ***V3B*** (peak value *t* = −5.61; at [1, −89, 12]; *k* = 792 vertices), ***V2*** (peak value *t* = −6.27; at [23, −102, −4]; *k* = 644 vertices), ***RI*** (peak value *t* = −6.25; at [−21, −2, −7]; *k* = 472 vertices), area ***6a*** (peak value *t* = −5.39; at [2, 18, 59]; *k* = 302 vertices), area ***2*** (peak value *t* = −4.60; at [−1, −31, 61]; *k* = 272 vertices), area ***6a*** including the area ***6ma*** belonging to the paracentral lobular and mid cingulate cortex (PLMCC), area ***s6–8*** belonging to dorsolateral prefrontal cortex (DLPFC) (peak value *t* = −5.43; at [6, 39, 53]; *k* = 236 vertices), ***POS2*** (peak value *t* = −4.64; at [28, −70, 17]; *k* = 229 vertices), ***V4*** (peak value *t* = −5.17; at [−3, −102, −19]; *k* = 213 vertices), and ***FEF*** (peak value *t* = −5.05; at [−11, 22, 49]; *k* = 210 vertices) at a *p-*value of <0.001 (corrected) at cluster level (see Figure 2A and Table 4). While no significantly increased CT was found in adolescent MDD.

**Table 4:** Locations of regional decreased CT at the left hemisphere in the MDD group.

**Region**		**MNI coordinates of peak**			**Peak *t*-value**	**Spatial extent (in contiguous vertices)**
**HCP-MMP1.label**	**Yeo2011_7Networks**	***x***	***y***	***z***		
*MDD < HC*						
**LIPd**	**7Networks_3**	−5	−53	42	−5.70	852
**V3B**	**7Networks_1**	1	−89	12	−5.61	792
**V2**	**7Networks_3**	23	−102	−4	−6.27	644
**RI**	**Medial wall**	−21	−2	−7	−6.25	472
**6a, 100%**	**7Networks_3**	2	18	59	−5.39	302
**Area 2**	**7Networks_2**	−1	−31	61	−4.60	272
**6a, 92.7%**	**7Networks_6**	6	39	53	−5.43	236
6ma, 3.4%	PLMCC					
s6–8, 3.4%	DLPFC					
**POS2**	**7Networks_7**	28	−70	17	−4.64	229
**V4**	**7Networks_1**	−3	−102	−19	−5.17	213
**FEF**	**7Networks_3**	−11	22	49	−5.05	210

#### 3.3.2. Decreased CT in adolescent MDD at the right hemisphere

In the right hemisphere, MDD patients also showed a significantly decreased CT in area ***6a*** including area ***FEF*** (peak value *t* = −5.49; at [0, 20, 56]; *k* = 554 vertices), ***V3B*** (peak value *t* = −5.93; at [−1, −87, 14]; *k* = 414 vertices), ***AIP*** (peak value *t* = −4.91; at [11, −29, 46]; *k* = 309 vertices), area ***6a*** including area ***i6–8*** and ***s6–8*** belonging to DLPFC (peak value *t* = −4.53; at [−5, 46, 49]; *k* = 303 vertices), ***V1*** (peak value *t* = −6.17; at [−22, −59, −15]; *k* = 239 vertices), ***7AL*** (peak value *t* = −5.32; at [−16, −51, 64]; *k* = 213 vertices), and ***V2*** (peak value *t* = −4.74; at [−26, −81, 5]; *k* = 145 vertices, respectively) at *p* < 0.001 (corrected) at the cluster level (see Figure 2B and Table 5). Moreover, no significantly increased CT was found in adolescent MDD.

**Table 5:** Locations of regional decreased CT at the right hemisphere in the MDD group.

**Region**		**MNI coordinates of peak**			**Peak *t*-value**	**Spatial extent (in contiguous vertices)**
**HCP-MMP1.label**	**Yeo2011_7Networks**	***x***	***y***	***z***		
*MDD < HC*						
**6a, 93.7%**	**7Networks_3**	0	20	56	−5.49	554
FEF, 6.3%						
**V3B**	**7Networks_1**	−1	−87	14	−5.93	414
**AIP**	**7Networks_3**	11	−29	46	−4.91	309
**6a, 53.8%**	**7Networks_6**	−5	46	49	−4.53	303
i6–8, 33.3%	DLPFC					
s6–8, 12.2%	DLPFC					
**V1**	**7Networks_1**	−22	−59	−15	−6.17	239
**7AL**	**7Networks_3**	−16	−51	64	−5.32	213
**V2**	**7Networks_1**	−26	−81	5	−4.74	145

### 3.4. Group comparison of ALFF/ReHo measurement of functional activity

#### 3.4.1. Increased ALFF in adolescent MDD

**Figure 3:**
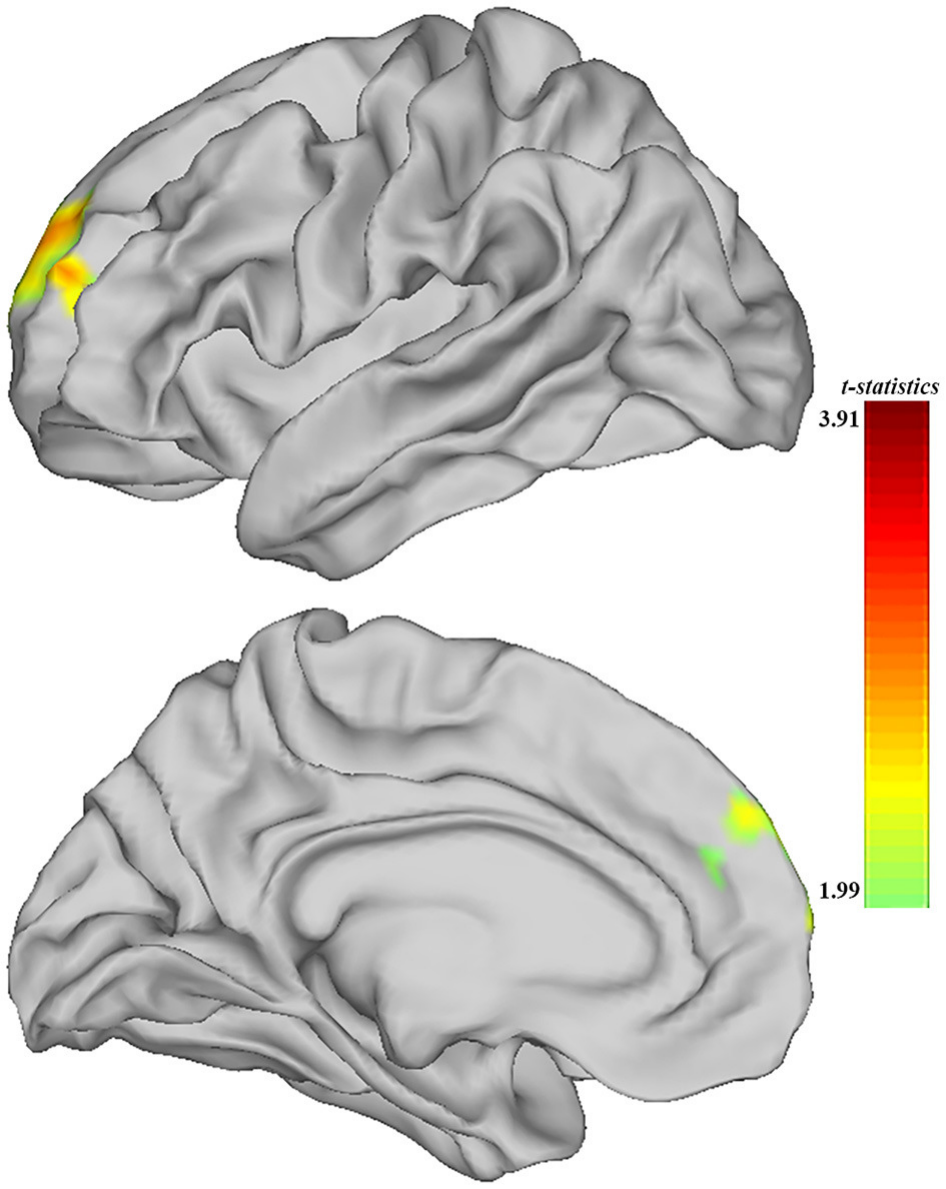
Adolescent MDD showed significantly increased ALFF of functional activation at ACC and DLPFC in the left hemisphere. The color bar depicts the *t*-statistics.

When comparing the ALFF measurement of functional activity, only a significant difference was observed in regional ALFF value at the surface space between the MDD and HC groups in the left hemisphere. MDD patients showed a significantly increased ALFF in ***9a*** belonging to the dorsolateral prefrontal cortex (DLPFC) and extending to ***9m*** belonging to the anterior cingulum (ACC) and medial prefrontal cortex (mPFC) (peak value *t* = 3.00; at [−23, 53, 20]; *k* = 97 vertices) at a *p-*value of <0.05 (corrected) at the cluster level (see Figure 3 and Table 6).

**Table 6:** Locations of regional altered spontaneous functional activation in the MDD group.

**Region**		**MNI coordinates of peak**			**Peak *t*-value**	**Spatial extent (in contiguous vertices)**
**HCP-MMP1.label**	**Yeo2011_7Networks**	***x***	***y***	***z***		
**ALFF**						
*MDD > HC*						
**9a**	**7Networks_7**	−23	53	20	3.00	97
9m	**7Networks_7**					

#### 3.4.2. No significant differences in ReHo in adolescent MDD

When comparing the ReHo measurement of functional activity, no significant differences were found between the two groups.

### 3.5. Group comparison of signal from the region of interests and correlation with clinical scores

**Figure 4:**
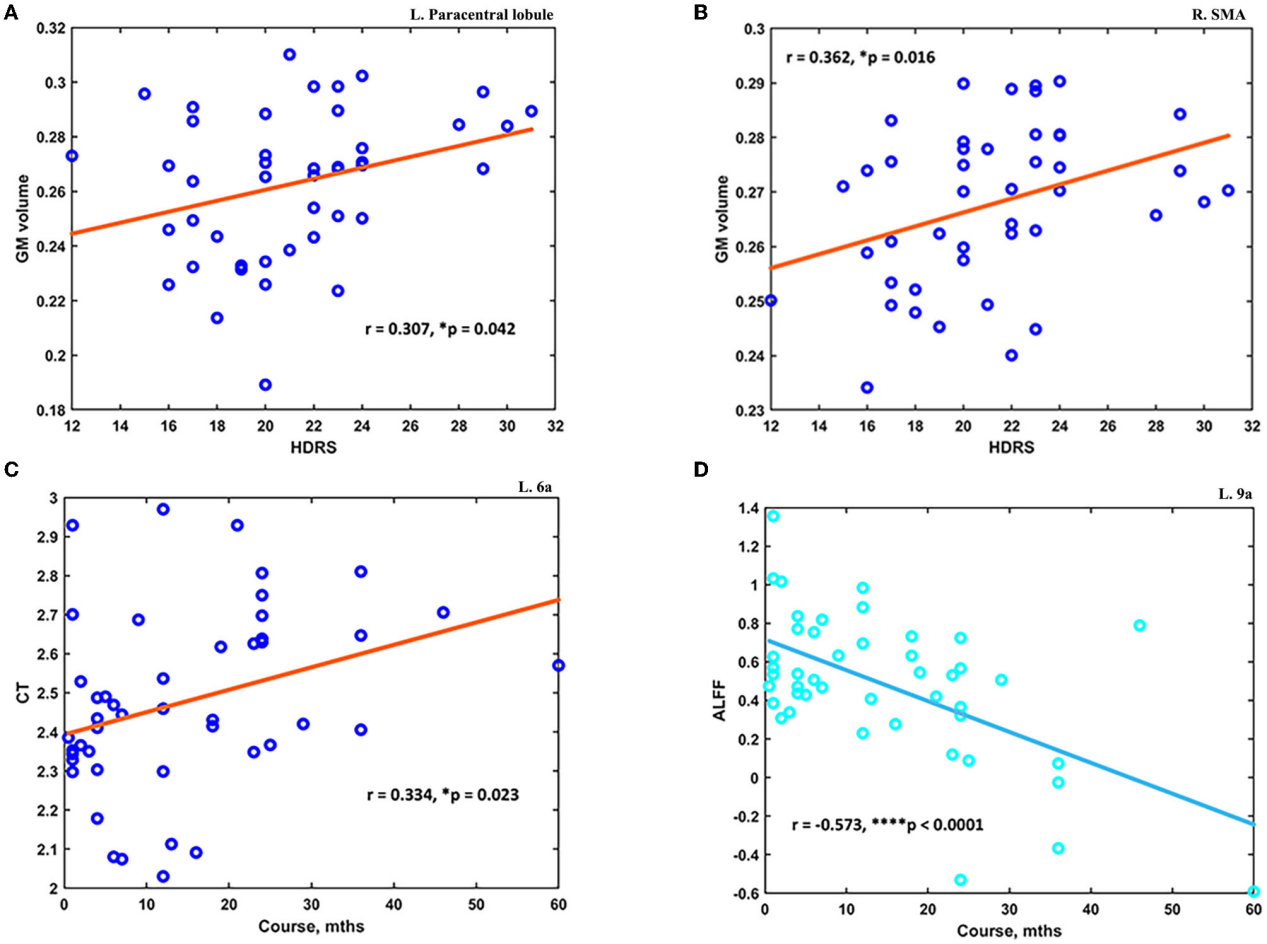
The altered measurements in adolescent MDD showed significant correlations with clinical scores. **(A)** The increased GM at the left paracentral lobule was positively correlated with HDRS scores (*N* = 44). **(B)** The increased GM at the right SMA was positively correlated with HDRS scores (*N* = 44). **(C)** The decreased CT value at the subdivision of the left superior premotor cortex was positively correlated with the course of MDD (*N* = 46). **(D)** The increased ALFF at left ACC and mPFC was found negatively correlated with the course of MDD (*N* = 46). ^*^p < 0.05; ^****^p < 0.0001.

The *post hoc* analyses showed significant differences in signal from altered brain regions reported before between the MDD and HC groups (see details in Supplementary material). The altered measurements of the GM volume at several brain regions were found to correlate with HDRS scores. First, the mean GM at the left paracentral lobule was significantly higher in MDD patients (0.263 ± 0.027) compared to HC (0.215 ± 0.041) and positively correlated with HDRS scores (*N* = 44; *r* = 0.307, *p* = 0.042) (see Figure 4A). Second, the mean GM at right SMA was significantly higher in MDD patients (0.268 ± 0.015) compared to HC (0.246 ± 0.018), and it was also found positively correlated with HDRS scores (*N* = 44; *r* = 0.362, *p* = 0.016) (see Figure 4B). Moreover, the altered measurements of CT and ALFF at several brain regions were found to correlate with the course of MDD. The mean CT at ***6a*** was significantly lower in MDD patients (2.490 ± 0.251) compared to HC (2.774 ± 0.213), and it was positively correlated with the MDD course (*N* = 46; *r* = 0.334, *p* = 0.023) (see Figure 4C). Finally, the ALFF at left ACC and mPFC was found significantly higher in MDD patients (0.462 ± 0.412) compared to HC (0.181 ± 0.341), and it was negatively correlated with the course (*N* = 46; *r* = −0.573, *p* < 0.0001) (see Figure 4D).

## 4. Discussion

The present study examined the neuroanatomical morphometry including regional GM volume and CT and also measured the functional activation through ALFF and ReHo in adolescent MDD. Compared to healthy controls, altered GM volumes were found at frontal–temporal–parietal and subcortical brain areas involved with various information processing in adolescent MDD. Furthermore, thinner CT was observed at cortices responsible for visual and auditory processing and motor movements in adolescent MDD. Moreover, hyperactivity indicated by ALFF was found at ACC and mPFC extending to DLPFC cortices in adolescent MDD. Correlation analyses across those altered structural and functional features showed that increased GM volume at the left paracentral lobule and right SMA was positively correlated with HDRS scores. In the meantime, the thinner CT at the subdivision of the left superior premotor cortex was found positively correlated with the course of MDD, while the hyperactivity at left ACC and mPFC was negatively correlated with the course.

### 4.1. Findings of neuroanatomical alterations and correlates in adolescent MDD

The inferior temporal cortex, the ventral pathway of the two major processing pathways conveying visual information, is crucial for the processing and storage of information about object detection (23, 24). The parahippocampus, located in the medial temporal, has been implicated in navigation and visual memory. A particular area within the parahippocampus named parahippocampal place area was found to represent places by encoding the geometry of the local environment, thus perceiving the local visual environment (25). The fusiform gyrus is a large region in the inferior temporal cortex that plays important roles in object and face recognition. The recognition of facial expressions is located in the fusiform face area, while bodies are selectively in the fusiform body area (26). A recent study has also reported increased functional connectivity between subdivisions of bilateral parahippocampal and right inferior temporal with frontal eye field in adolescent MDD (27). Thus, the increased GM volume found here might be suggestive of abnormal visual information processing that existed in an adolescent with depression.

The left mTG is within the neural pathway in the processing of both concrete and abstract words, underpinning the brain language understanding function (28). A recent meta-analysis regarding the neural correlates of neuroticism, an important factor for the development of MDD, has reported that spontaneous activity in the left mTG was positively correlated with neuroticism. It could reflect the negativity and instability of a highly neurotic individual's emotional experience (29). The orbIFG is closely connected with the lateral orbitofrontal cortex. The left orbIFG was reported higher activation to blend aromatic mixtures components in an odor configural experiment, implying its role as a mediator of configural percepts between temporal and orbitofrontal areas involved in configural memory processes (30). The caudate nucleus, a nucleus of the basal ganglia, is demonstrated to represent action-outcome contingencies sub-serving adaptable goal-directed behavior (31). The altered GM volume might suggest dysfunctional mediation and adaptation in adolescent MDD.

Evolved first as a motor–control region aligned with the sensory integration of olfactory-guided group behavior in mammals, the insula evolved later for cortical processing of homeostatic sensory activity in the individual animal. The insula is implicated in a wide range of conditions and behavior such as interoception, recognition, emotional awareness, decisions, cognitive control, and performance monitoring. The primary interoceptive representation of the physiological condition of the body is in the posterior insula, whereas the anterior insula contains interoceptive representations that substantialize all subjective feelings from the body and perhaps emotional awareness (32). In addition, it is part of an extended salience network, which is involved in the bottom–up detection of salient stimuli, interoceptive awareness for positive and negative internal states, and switching between emotional brain areas and more central executive regions. Insula abnormalities may reflect a disproportionate allocation of resources to the internal experience of negative self-focus thinking and emotional experience and a failure to switch to higher-order cognitive processes involved in the reappraisal of negative emotions and in allocating toward the external environment, which may, in turn, contribute to the development of MDD. The altered GM volume found here might reflect such negative self-focus thinking in adolescent MDD.

The paracentral lobule is a U-shaped convolution that loops below the medial part of the central sulcus and includes the motor and sensory areas for the lower limbs. The SMA is a portion of the premotor cortex located on the medial surface of the cortex anterior to the precentral sulcus. Consistent with our findings, a previous study has reported regional cerebral blood flow in the precentral gyrus in unmedicated first-episode MDD adolescents (33). As a key part of the medial premotor system, the SMA is suggested to play an important role in the development of the intention-to-act and the specification and elaboration of action through its mediation between the medial limbic cortex and the primary motor cortex (34). Thus, the positive correlation between the GM volume and depression severity might suggest the potential for paracentral lobule and SMA to serve as neural markers of depression in adolescents.

The thalamus, the largest subdivision of the diencephalon, plays an indispensable role in the modulations of messages involved in corticocortical processing (35). As it relays almost all sensory information except the olfactory, the thalamus is involved in the process of functional activities such as sleep, memory, and motor. Moreover, through the regulation of hormone generation and release, the hypothalamic could regulate the neurovegetative systems. Therefore, the decreased GM volume at the thalamus might be the underlying neural mechanism of impaired information processing and neurovegetative symptoms in adolescent MDD. The decreased GM volume at MFG and SFG has been reported in previous studies on first-episode, medication-naive adult MDD patients (36–38). The volumetric differences of left MFG were found to be predictive of individual differences in cognitive control capacity (39), thus, suggesting its role in cognition processes. In line with the structural alteration, depressive patients were found to have a significantly lower response to reward stimulus in the MFG (40). The deficits of the GM volume in MDD patients implied dysfunctional cognitive and emotional control. Furthermore, the circuit composed of the medial dorsal thalamus and mPFC was identified to control depression-like behavior, and synaptic regulation of this thalamocortical circuit could elicit a decrease in despair-like behavior (41).

Hippocampus, located between the thalamus and medial temporal cortex, belongs to part of the limbic system. Converging evidence showed its role in the pathophysiology of MDD. The elevated glucocorticoid levels associated with MDD may negatively affect neurogenesis, causing excitotoxic damage or further key neurotrophins in the hippocampus (42). The cuneus is the gyrus immediately superior to the calcarine sulcus. It encompasses the primary visual cortex, the region receiving thalamocortical connections from the lateral geniculate nucleus of the thalamus. The left cuneus GM volume was found significantly associated with working memory performance (43). Thus, the decreased GM volume at those two structures might be the neural basis of impaired memory in adolescent MDD.

The ACC forms the anatomical circuit mediating the motivated behavior, and damage to this area would generate apathy (44). Patients are rarely moving, incontinent, drink, and eat only when fed, and if speech occurs, it is limited to monosyllabic responses to others' questions. Furthermore, as subdivisions of the cingulum, the ACC is argued to be the affective division, and MCC to the cognitive division. The negative affect and cognitive control were reported to activate those anterior midcingulate cortex, and the areas constitute a hub where information can be linked to motor centers responsible for expressing affect and executing goal-directed behavior (45). Thus, decreased GM in those regions might impact the affective and cognitive processing in adolescent MDD. Furthermore, the postcentral and precentral gyrus are the somatosensory and motor cortex, which are responsible for sensorimotor interactions. The reduction of GM in those areas might suggest sensory and motor function deficits in adolescent MDD. Located in the inferior parietal, the supramarginal gyrus, especially the right side, is essential for visuospatial awareness. It was also found to have a negative correlation with neuroticism, and this area could help link the perception of socio-affective stimuli to emotions (29). The decreased GM found might imply the decreased awareness and perception in adolescent MDD.

The broad lower CT was found at the visual, motor, and superior parietal cortex at both hemispheres of the brain in adolescent MDD. V1 is the primary visual cortex, which is proposed to provide a saliency map with V1's output neurons firing rates increasing monotonically with the salience value of the visual input under a given visual scene (46). Furthermore, together with the early visual cortex, the activity in V1, V2, and V3 is demonstrated to sustain during the maintenance of attention in the absence of visual stimulation (47). The dorsal and ventral stream visual cortex commonly contribute to shape perception, and location processing was suggested to be essentially a function of the dorsal visual pathway (48).

Frontal eye fields, located anterior to area 4, are the origination of one of the five parallel neuroanatomical circuits implicated in psychiatric disorders (47). The primary motor cortex was suggested to share the role in the control of libs movement coordination with SMA (49). The premotor cortex was considered to play a role in the perception of speech, providing an internal motor simulation of the perceived phonemes (50). Lower CT was found at two subregions of the *6a* at both the left and right hemispheres. The first cluster peaked at *6a* and contains the areas belonging to the dorsal attention network, which is concerned with the orientation of one's focus to a specific task (51). The second cluster peaked at *6a* and contains subareas supporting cognitive control and decision-making processes (e.g., PLMCC, DLPFC) belonging to the frontoparietal network. Containing the subregions of the same *6a*, the frontoparietal network is anatomically positioned between components of the dorsal attention, and the hippocampal-cortical memory systems for information integration (52). The lower CT found at those two *6a* subregions might suggest the diminished cognitive processes in adolescent MDD. Furthermore, the positive correlation at the subregion belonging to the frontoparietal network could suggest such neurocognitive processing underlying adolescent MDD. Located between the two major sensory modalities of visual and somatosensory domains, the superior parietal cortex forms a bridge between them. The reduced CT found in those regions might suggest decreased visual attention and motor movements in adolescent MDD.

### 4.2. Findings of functional alterations and characteristics in adolescent MDD

DLPFC and ACC are the central nodes in the circuits mediating motivated behavior and executive control, respectively (44). The previous study reported that greater emotion dysregulation in school-age predicted alterations in connectivity between DLPFC and dorsal ACC in children with a history of depression (53). The higher brain activity in the DLPFC measured by ALFF was also reported in adolescent MDD (54). The reduced DLPFC in response to negative social status explained the positive correlation between self-reported social risk and depressive symptoms in youth, implying the DLPFC underlying the neural substrate of cognitive processing for emotion (55). In the same trend as our findings, adolescents with MDD were found to exhibit a higher connection of DLPFC and ACC with the subcortical insula (18). The increased activity found at ACC and DLPFC, and its negative correlation with MDD course might explain the rumination, impaired concentration, and physiological arousal in adolescent MDD.

The current study examined the neuroanatomical morphometry and functional activation alterations in adolescent MDD through volumetric and surface-based analyses. Broader changes in measurements were observed in cortico-subcortical brain areas. Some limitations existed in the current study. First, the findings were based on a sample size of 50 MDD patients, a large sample size could be carried out in the future to validate and replicate the findings. Furthermore, women were reported to be about two times as likely as men to develop depression and may have different clinical symptoms (56). Therefore, future studies with a large number of samples could be carried out to examine the alterations in women and men, respectively. Moreover, due to the limited records, we could not carry out the examinations of these measurements alterations' correlation with clinical symptoms. Thus, future studies with full-scale assessments of clinical severity or symptoms would benefit the interpretations. Moreover, growing evidence has shown DLPFC as a core brain hub of self-regulation, and it was newly reported to be the neural marker of grit, a psychological trait of perseverance and passion to pursue long-term goals (57). The altered neuronal spontaneous activity at DLPFC in adolescent MDD was found in this study, and it would be interesting to look into how its functional connection with other brain areas in future studies. Finally, MDD patients have a risk of suicide, previous studies have reported differences including decreased nodal efficiency of the GM network in the frontosubcortical circuit, and greater activity at ACC-DLPFC cortical attentional control circuitry in MDD patients with suicidality compared to MDD patients without suicidality (58, 59). Future studies would also examine the differences in adolescent MDD.

## 5. Conclusion

Combined MRI and fMRI, we demonstrated the morphometric and functional metrics alteration in adolescent MDD. Through whole-brain analyses, we detected GM volume changes in broader frontal–temporal–parietal and subcortical brain areas. Specifically, the increased GM volume at the left paracentral lobule and right SMA was positively associated with depression severity. Furthermore, we found a lower CT in brain areas for visual and auditory processing as well as motor movements, and the subdivision of the superior premotor area was positively correlated with the MDD course. It suggested decreased visual attention and motor activity in adolescent MDD. Moreover, the functional activity measured by ALFF was found to be increased at ACC and mPFC, and this hyperactivity was negatively correlated with the course of MDD. It might suggest that rumination, impaired concentration, and physiological arousal existed in adolescent MDD. Altogether, our findings provided evidence of neuroanatomical and spontaneous functional activity disturbances in adolescent MDD and might help shed light on the underlying pathophysiology.

## Data availability statement

The raw data supporting the conclusions of this article will be made available by the authors, without undue reservation.

## Ethics statement

The study was approved by the research Ethics Committee of the Chongqing Medical University. Written informed consent to participate in this study was provided by the participants' legal guardian/next of kin.

## Author contributions

XZ: conceptualization, formal analysis, visualization, funding acquisition, writing—original draft, and writing—reviewing and editing. JCa: data curation and funding acquisition. QH: data curation and formal analysis. SH and LD: resources. XC, JCh, MA, YG, and JH: writing—reviewing and editing. LK: project administration, funding acquisition, and writing—reviewing and editing. All authors have approved the final manuscript.
