# Supplementary material for: Severity related neuroanatomical and spontaneous functional activity alteration in adolescents with major depressive disorder
Source: Front Psychiatry. 2023 Apr 5;14:1157587. doi: 10.3389/fpsyt.2023.1157587 (PMC10113492; doi:10.3389/fpsyt.2023.1157587)
Supplement: Supplementary file 1 [file Data_Sheet_1.docx]

Supplementary Material

# Supplementary Figures

The post-hoc analyses across mean signal of regional neuroanatomical and functional alterations showed significant differences between the adolescent MDD and HC groups (see the following figures for details).

**
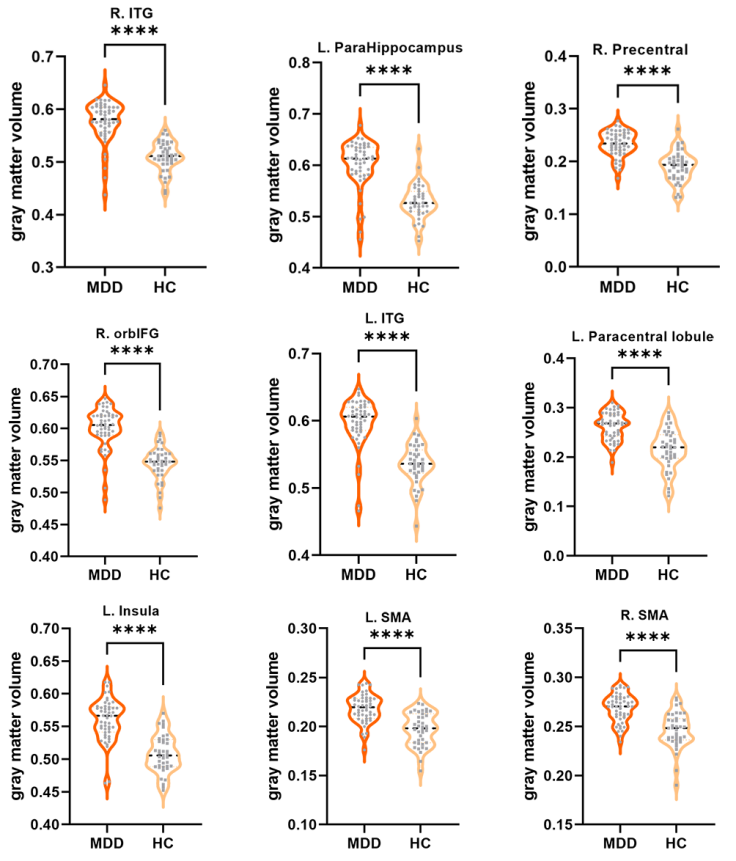
**

**Supplementary Figure 1.** The group comparison of mean GM volume at brain regions showing significantly increased GM volume in adolescent MDD compared to HC (^****^ indicates p < 0.0001).


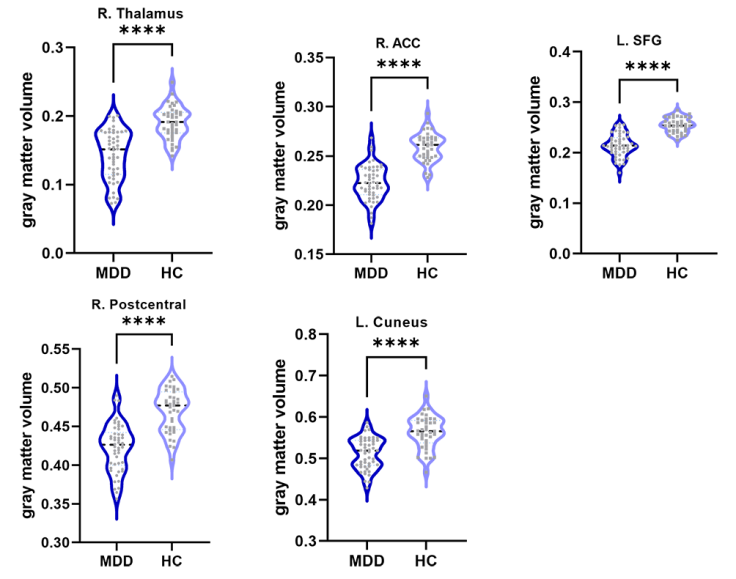


**Supplementary Figure 2.** The group comparison of mean GM volume at brain regions showing significantly decreased GM volume in adolescent MDD compared to HC (^****^ indicates p < 0.0001).


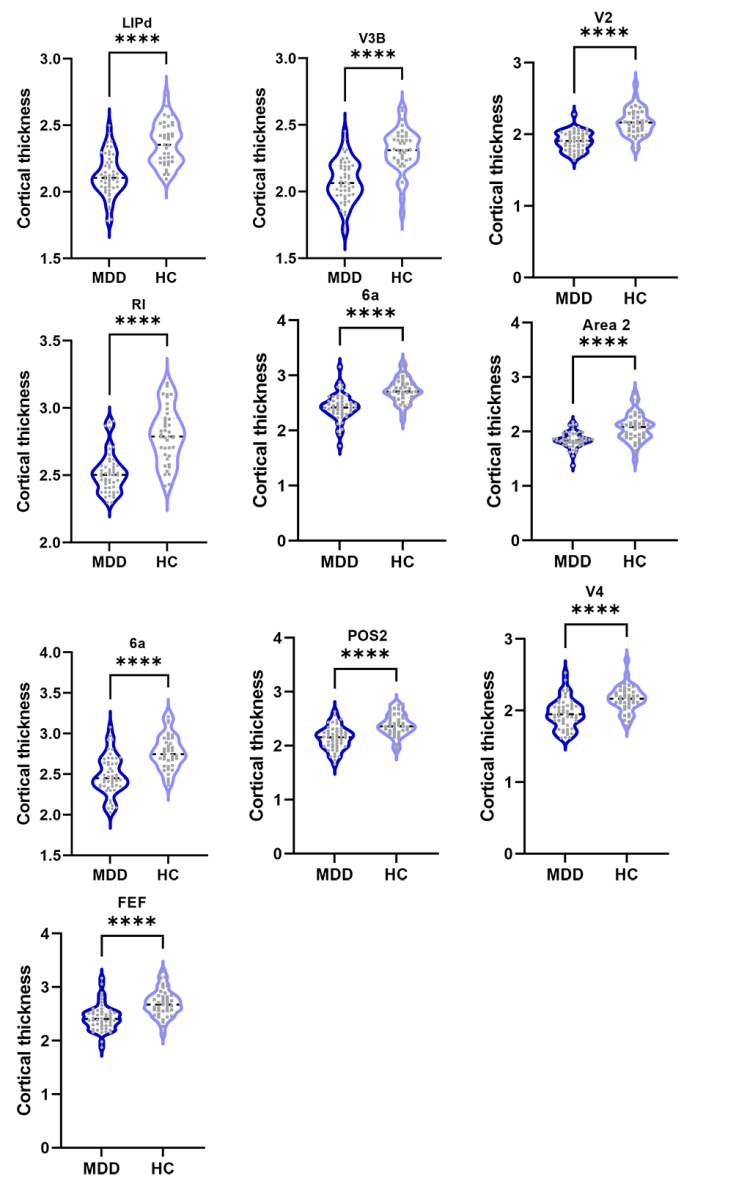


**Supplementary Figure 3.** The group comparison of mean CT at brain regions of left hemisphere showing the significantly lower value in adolescent MDD compared with HC (^****^ indicates p < 0.0001).


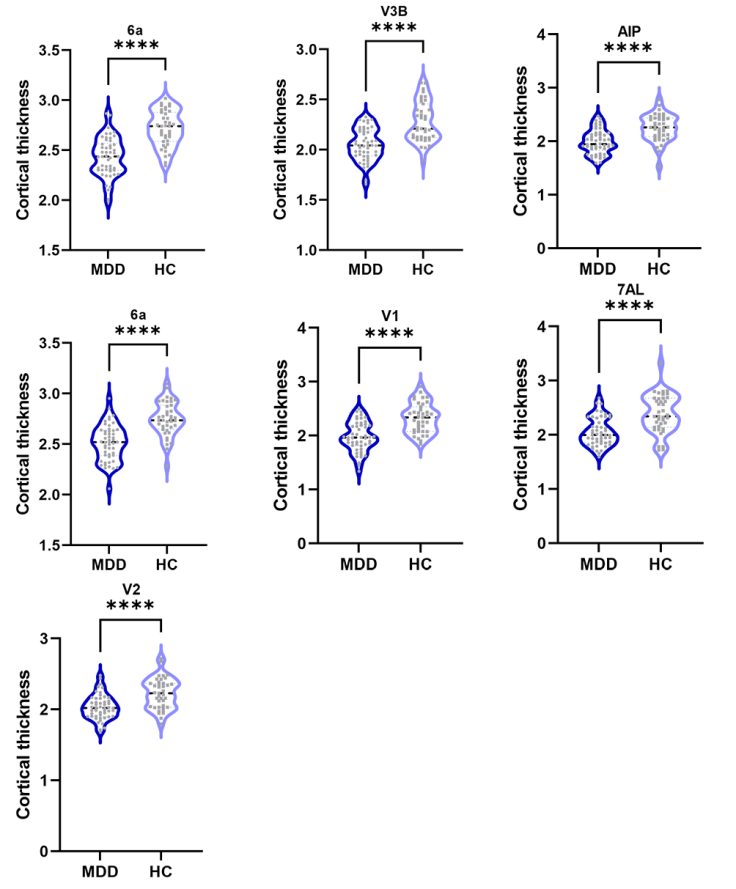


**Supplementary Figure 4.** The group comparison of mean CT at brain regions of right hemisphere showing the significantly lower value in adolescent MDD compared to HC (^****^ indicates p < 0.0001).


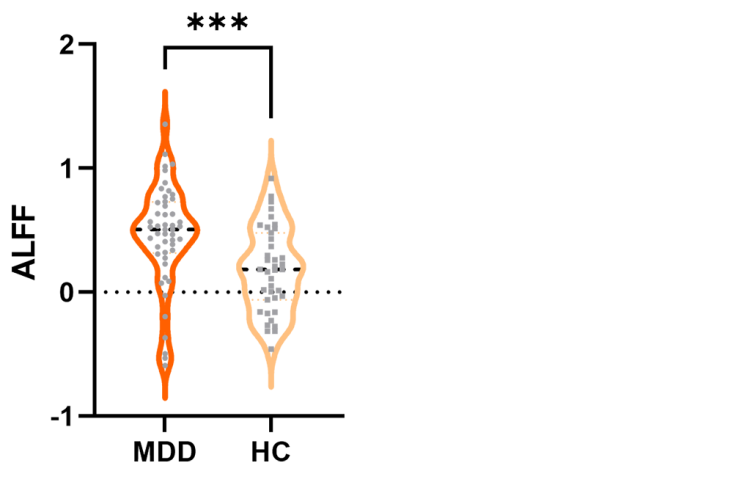


**Supplementary Figure 5.** The group comparison of mean ALFF at brain regions showing significantly higher regional spontaneous neuronal activity in adolescent MDD compared to HC (^***^ indicates p < 0.001).
